# Awakening the *kundalini* of humour

**DOI:** 10.4103/0019-5545.31565

**Published:** 2006

**Authors:** Harish Shetty

**Affiliations:** * Social Psychiatrist, 4A/11, Taxilla, Mahakali, Mumbai 400093, Maharashtra; e-mail: harsha139@yahoo.co.in

While conducting training sessions for judicial officers on mental health for the past three years I would always ask them a question: ‘Why is it that they do not smile?’ The reply I always got was that ‘It will be misunderstood and trivialize such a responsible activity.’

Not very long ago, when I was conducting a session for senior medical teachers in a reputed teaching hospital, an orthopaedic surgeon shared that he would never smile at a patient in pain as the latter would feel offended. My question to him was: ‘If your close relative knocks at your door to seek help for some aches and pains, will you not greet him with a smile?’ Humour and its brethren (i.e. smile/laughter/a naughty giggle) all contribute to healing. As the recipient feels better, the giver feels ecstatic.

Humour is a lost art and the casualty of a fast-paced world. I believe that the day should begin with humour. As far as possible, I begin my day with laughter and it could be with anybody. Well, the auto rickshaw driver could be nudged by *‘Chal Dhanno’.* Many a time the drive is recklessly dangerous but the point is that the human connection is established. This bond is losing its shine as doctors have forgotten this basic need under the belt of effective molecules or therapies.

Swaminath has made this point very effectively. I remember the session with a bunch of students in a hostel recently following the suicide of their colleague. As the session moved very seriously with tears and fears expressed, one little girl shared a joke sent through sms. Nervous smiles erupted and as I nodded a subtle sanction, more jokes flowed with no guilt or apprehension. Deeper sharing erupted and the kids moved ahead in their process of healing.

In 1993, after the devastating earthquake at Latur (Maharashtra) we had organized *a Manaswasthya Shibir—a* mental health camp. This session was organized in the famous Neelkanteshwar temple at Killari which was also partially devastated. We had just began our ‘funda’ with our PTSD business, when the villagers cracked a joke on their own. As they continued, the session became hilarious and we fulfilled our agenda. The fantastic component was that the villagers set the agenda and the method of healing through their own cultural and mental processes. Most of my teachers have been grim, racking their brains to differentiate between the different types of delusions. Yet all of them were so different with a glass of whisky. I always wonder why they could never reveal this side as clinicians in their day-to-day life. Why were their chats with patients so formal, so cold, so clinical? The flesh and bones were dissected by a set of questions evolved by many OCPD clinicians.

Sexual innuendos alone are not humour. And compartmentalizing it during different times for self-conceived reasons is like going away from God—moving away from Mother Nature. As I smile at the cops on the road chatting away with my ‘Lord Krishna’ (the guy who drives me around), I was stopped by one. My ‘Lord Krishna’ came back perplexed after showing him the car papers. When questioned he shared, ‘Your boss is laughing on the street where no one does. Surely his papers are not in order.’ Mistrust kills humour and it kills the being in you, the life *chetna* with which every human being is endowed.

The revival of humour in teaching and practice is the need of the hour. This is not necessarily aimed alone to humanize therapies but to humanize the therapist and make psychiatry relevant to all beings and not only to a few receptors. Teachers who cannot laugh during their work need to quit and be morgue attendants. Well, they also ought to laugh.

Swaminath speaks about the importance of beginning with a humorous tone but I get foxed by an observation in my clinic. I share my clinic with a physician who is shy, wry and honest. He has a great practice and most of his patients respect him as he is serious and is thus inferred as committed. My analysis suggests that probably humour has to be in a measured dose at the right time and at the right place. After my experience at Latur, I have evolved a strategy for my training workshops. The *mantra* for a disaster worker is that when a survivor initiates laughter go with it but do not jump on the bandwagon and be a driver. Let him/her be and you gently nudge the process with all participants as partners in the process.

‘You are so serious’ is the comment I receive from patients on my bad days and they empathize with me when I share that I am not in a very good mood and would examine them in detail during their next visit if it can wait. The vulnerability of a doctor is still in the zone of denial. Its acceptance is the key to be at ease and make every cell dance in our body.

The triad of feelings/adjectives/sarcasm and their inter-relationships is the key to aid release and create humour. The maxim that originated in my persona during a bout of humour is the bottom-line for me: ‘Feelings are like food items. They would decay on delay.’ Those who survive on the plane of feelings retain humour and those who live on the mountains of adjectives and sarcasm do not realize that the delay in expressing feelings would kill the laughter.

Swaminath's piece is hilarious, with effortless ease he helps discover our forgotten software. These programs are priceless and buried as Egyptian pyramids in the sands of time. Let us awaken our *kundalini* of humour which has no patent and is not governed by the W.T.O.

